# Do patient decision aids help people who are facing decisions about solid organ transplantation? A systematic review

**DOI:** 10.1111/ctr.14928

**Published:** 2023-02-19

**Authors:** Georgina L. Irish, Alison Weightman, Jolyn Hersch, P. Toby Coates, Philip A Clayton

**Affiliations:** ^1^ Faculty of Health and Medical Science University of Adelaide Adelaide Australia; ^2^ Australia and New Zealand Dialysis and Transplant (ANZDATA) Registry South Australian Health and Medical Research Institute (SAHMRI) Adelaide Australia; ^3^ Central and Northern Adelaide Renal and Transplantation Service Royal Adelaide Hospital Adelaide Australia; ^4^ School of Public Health Faculty of Medicine and Health The University of Sydney Sydney Australia

**Keywords:** organ transplantation, patient decision aid, systematic review

## Abstract

**Background:**

Decisions about solid organ transplantation are complex. Patient decision aids (PDAs) enhance traditional education, by improving knowledge and supporting patients to align their values with treatments. There are increasing numbers of transplantation PDAs, however, it is unclear whether these are effective. We conducted a systematic review of studies assessing the impact of PDA use in transplantation.

**Methods:**

We searched the Cochrane Register of Controlled Trials, CINAHL, EMBASE, MEDLINE, and PsycINFO databases from database inception to October 26, 2020. We included primary studies of solid organ transplantation PDAs defined by the International Patient Decision Aids Standards. All comparators and reported outcomes were included. Mean difference in knowledge (before vs. after) was standardized on a 100‐point scale. Pooled‐effect for PDAs was calculated and compared to the standard of care for randomized controlled trials (RCTs) and meta‐analyzed using random effects. Analysis of all other outcomes was limited due to heterogeneity (PROSPERO registration, CRD42020215940).

**Results:**

Seven thousand four hundred and sixty‐three studies were screened, 163 underwent full‐text review, and 15 studies with 4278 participants were included. Nine studies were RCTs. Seven RCTs assessed knowledge; all demonstrated increased knowledge with PDA use (mean difference, 8.01;95%CI 4.69–11.34, *p* < .00001). There were many other outcomes, including behavior and acceptability, but these were too heterogenous and infrequently assessed for meaningful synthesis.

**Conclusions:**

This review found that PDAs increase knowledge compared to standard education, though the effect size is small. PDAs are mostly considered acceptable; however, it is difficult to determine whether they improve other decision‐making components due to the limited evidence about non‐knowledge‐based outcomes.

## 1 INTRODUCTION

Solid organ transplantation is the best treatment option for most people with solid organ failure.^1^, ^2^, ^3^, ^4^, ^5^, ^6^, ^7^ For some organs, such as heart, lung, or liver transplants, the choice is often between transplantation and conservative care. The decisions for kidney and pancreas transplants are more nuanced as there are other life‐sustaining treatments beyond transplantation. For most patients, transplantation offers a survival benefit and improved quality of life.^8^, ^9^, ^10^, ^11^, ^12^ However, the degree of benefit for a transplant recipient varies.^2^, ^4^, ^13^, ^14^, ^15^, ^16^ Additionally, any benefit, either for survival or quality of life, must be balanced against the risk of transplant‐associated harms.^17^, ^18^ Therefore, these decisions can be difficult^19^, ^20^, ^21^ and are different for everyone. To decide which option is best for them, patients must have adequate knowledge about treatment options and align their values with their risk‐benefit profile.

Patient education is the process of knowledge transfer, to allow recipients to make an informed decision about health treatments.^22^ Transplantation education must impart knowledge and explore the risk‐benefit profiles of different options in an individualized way.^20^, ^21^ Treatment decisions require balancing of rational and emotional assessments of benefits and risks, thus decision‐making needs to address knowledge as well as individual patient concerns.^21^, ^23^, ^24^ Patients with organ failure are not as informed as they would like about transplantation, despite being motivated to be involved in treatment decisions.^25^, ^26^, ^27^, ^28^, ^29^

Patient decision aids (PDAs) are tools to communicate evidence‐based information about the benefits and harms of different healthcare options.^30^ Their purpose is to meet the two key components of shared decision‐making by imparting information plus aligning patient values with potential treatments. PDAs are tools that can be used to supplement patient‐provider discussions and may assist with both components of decision‐making. In other healthcare fields, a large Cochrane review demonstrated PDAs increase knowledge and improve congruence with patients’ values.^31^ Despite this, the effectiveness of PDAs for transplantation has not been systematically analyzed, thus it is unclear whether they are effective in this field. There are an increasing number of PDAs for organ transplantation including some in current use.^32^, ^33^, ^34^, ^35^, ^36^, ^37^, ^38^, ^39^, ^40^, ^41^, ^42^, ^43^, ^44^, ^45^, ^46^, ^47^ It is therefore necessary to assess whether PDAs are effective for knowledge and decisions about organ transplantation.

## 2 MATERIALS AND METHODS

We performed a systematic review of all studies of PDAs in solid organ transplantation evaluating all outcomes, including a meta‐analysis of randomized controlled trials (RCTs) assessing knowledge. The study was conducted based on the Cochrane Handbook for Systematic Reviews on Interventions.^48^ This review complies with the Preferred Reporting Items for Systematic Review and Meta‐Analysis (PRISMA)^49^ guidelines. The review was registered with the database of prospectively registered systematic reviews in health and social care (PROSPERO):CRD42020215940.

**Practitioner Points**

1. Patients with organ failure face many complex decisions, especially relating to transplantation. There are increasing numbers of Patient Decision Aids (PDAs) available to assist decision‐making in solid organ transplant therefore it is important to clarify whether they are a useful adjunct.
2. There were no previous systematic reviews focused on PDAs for solid organ transplants, so it was unclear whether these tools increase knowledge or improve decision quality compared to standard education for this unique domain.
3. This review demonstrates that PDAs increase knowledge though the effect size is small. This supports the ongoing use of PDAs in this field. More work is needed to assess the impact of these tools on other measures of decision quality, as these were too heterogenous and infrequently assessed to draw meaningful conclusions.

### 2.1 Eligibility criteria

We included any studies of PDA use in solid organ transplantation. For comprehensiveness, we assessed any PDA used in any setting, in any country, with any adult population.

We defined PDAs based on the IPDAS guidelines^50^:

- “The decision that is being considered is explicitly stated;
- The PDA provides evidence‐based information about a health condition, particularly the options, benefits and harms, probabilities, and uncertainties;
- The PDA helps patients to recognize that the decision is value sensitive and to clarify the values they place on the harms and benefits.”

The inclusion criteria were adults involved in decisions about solid organ transplantation, including living donors, recipients (living and deceased organs), carers, or clinicians. We included all comparators to the PDA. We included pre‐test/post‐test, nonrandomized, RCT, and pilot studies. There were no publication date, language, or publication‐status restrictions. Exclusion criteria were reviews and studies lacking a comparison, intervention, or outcome assessment. We excluded any studies for interventions that did not meet the definition of PDAs based on the IPDAS criteria during full‐text review.^50^ We contacted authors to access the PDA, if it was not freely available or described in enough detail to assess if it met this definition.

### 2.2 Search

The following databases were searched on the October 26, 2020.

- Cochrane Central Register of Controlled Trials
- Cumulative Index to Nursing and Allied Health Literature (CINAHL)
- Embase
- Medline
- PsycINFO

Unpublished studies were searched for via a grey literature strategy on the December 18, 2020. Sources were PDA repositories,^51^, ^52^ registries of clinical trials,^53^, ^54^, ^55^ clinical practice guidelines,^56^, ^57^ internet search engines (Google, Google Scholar), references of review articles,^31^, ^58^, ^59^, ^60^ and references cited in the included studies. The search strategy is outlined in Tables S1–S5. All studies were imported to COVIDENCE^61^ for screening. COVIDENCE is software, supported by the Cochrane Network, which facilitates concurrent screening, review, and analysis of manuscripts by multiple reviewers.^61^

### 2.3 Selection

All titles and abstracts were independently screened by two authors (G.I., A.W.). Full‐texts of relevant studies were reviewed for eligibility. Any disagreements were resolved by third‐reviewer consensus or discussion (P.C., J.H.). When multiple reports of the same study were found, the information extracted was collated and treated as one study.

### 2.4 Data collection process

Data were extracted from each study using the data collection forms via COVIDENCE^61^ extraction form 1.0. All data were extracted in duplicate by independent reviewers (G.I., A.W.). When the information was unavailable or unclear, authors were contacted for further details, on two occasions, 4 weeks apart. This included acquiring access to the PDA if it was not freely available. Data‐items collected included the study sample population, eligibility criteria, methods, intervention, comparator, and any outcome measures. The interventions were evaluated using the Standards for UNiversal reporting of patient Decision Aid Evaluation studies (SUNDAE) checklist.^30^ The SUNDAE checklist was developed by the IPDAS Collaboration to ensure that PDA evaluation studies are understandable and explain the components of the PDA. Any outcome measure assessed at any time point was included.

### 2.5 Study risk‐of‐bias assessment

Different risk‐of‐bias assessments were performed depending on the study type. Only one tool was used per study type. All RCTs were assessed using the ROB‐2 tool.^62^ Non‐randomized studies were assessed using the ROBINS‐I tool.^63^ Pre‐test/post‐test intervention studies were assessed for risk‐of‐bias using the National Institutes of Health Quality Assessment tool for before‐after interventions.^64^ This score has been used in other pre‐test/post‐test risk‐of‐bias assessments.^65^, ^66^ Questions 11‐12 in the National Institutes of Health Quality Assessment tool for before‐after interventions were completed but not reported as they did not apply to this intervention. All risk‐of‐bias assessments were undertaken by two reviewers with disagreements resolved by consensus. Risk‐of‐bias was done using software: COVIDENCE and ROB‐2 Excel macro. Risk‐of‐bias graphics were presented using the ROBVIS tool.^67^

### 2.6 Mean difference in knowledge for RCT

Knowledge was assessed differently depending on the type of study. For the RCT studies, the mean difference in patient knowledge before and after PDA use was compared to the mean difference in patient knowledge before and after standard of care (traditional education used at the transplant centers). All knowledge tests were developed by the primary authors and based on information within the PDA, so differed for every study.

### 2.7 Outcome measurement

All studies that measured knowledge did so shortly after the use of the PDA. The mean difference in knowledge, between baseline and shortly after either PDA or standard education use, was calculated. If these raw data were not given, then they were estimated from the graphs from the published studies. To compare the different studies, the proportion of accurate responses was scaled to be a standardized score from 0 (no knowledge) to 100 (perfect knowledge). This technique has been employed in other systematic reviews of PDAs.^31^ If no standard deviations (SD) were given but confidence intervals or *p*‐values were available, then the SDs were derived.^48^

### 2.8 Synthesis methods

The mean difference in knowledge was combined across the RCT studies using a random‐effects model because of the likelihood of differences in treatment effect due to intervention variability of the studies. The inverse variance method was used for meta‐analysis using RevMan.^68^ One study Waterman 2019^46^ had two intervention arms to one control group; to prevent counting the control group twice (unit‐of‐error analysis) the control arm was split in half so the control arm contributed to both interventions.^69^ The robustness of the results was assessed using sub‐group sensitivity analysis of different organs, different PDA formats (paper, web‐based) and excluding high risk‐of‐bias. Reporting bias was assessed by funnel plot.

### 2.9 Certainty assessment

We used the GRADE approach for certainty assessment which is considered best practice for assessing synthesized findings for systematic reviews.^70^ Only the mean knowledge difference assessed by RCT was suitable for GRADE assessment. Other outcomes were not able to be assessed using the GRADE guidelines as there were too few studies using the outcomes and they were assessed in different ways.

### 2.10 Knowledge assessment: Pre‐test/post‐test and non‐randomized studies

For pre‐test/post‐test studies and non‐randomized studies, meta‐analysis is not advisable as there is no control group to compare the outcome to. The summary of the effect estimate was performed using Cochrane methodology. Difference in mean knowledge before/after PDA use and statistical significance was documented in tabular‐form. For the non‐randomized study of knowledge, the outcome was tabled.

### 2.11 Other outcomes

For comprehensiveness, all outcomes in any study included were assessed as part of this review. The outcomes reported were; acceptability, accuracy of risk perception, adverse effects, behavior, choice made, communication, decisional conflict, durability of a decision, feeling informed, readiness, self‐efficacy, and value congruence. These were mapped to the IPDAS criteria.^71^ The definition and methods for synthesis are described in the Supplementary Appendix. All of these outcomes were too heterogenous to allow for quantitative synthesis so qualitative summary synthesis was used. Meta‐analysis was not feasible for any outcomes apart from knowledge due to differing tools for assessment and outcomes not having an RCT control arm.

## 3 RESULTS

### 3.1 Study selection

**FIGURE 1:**
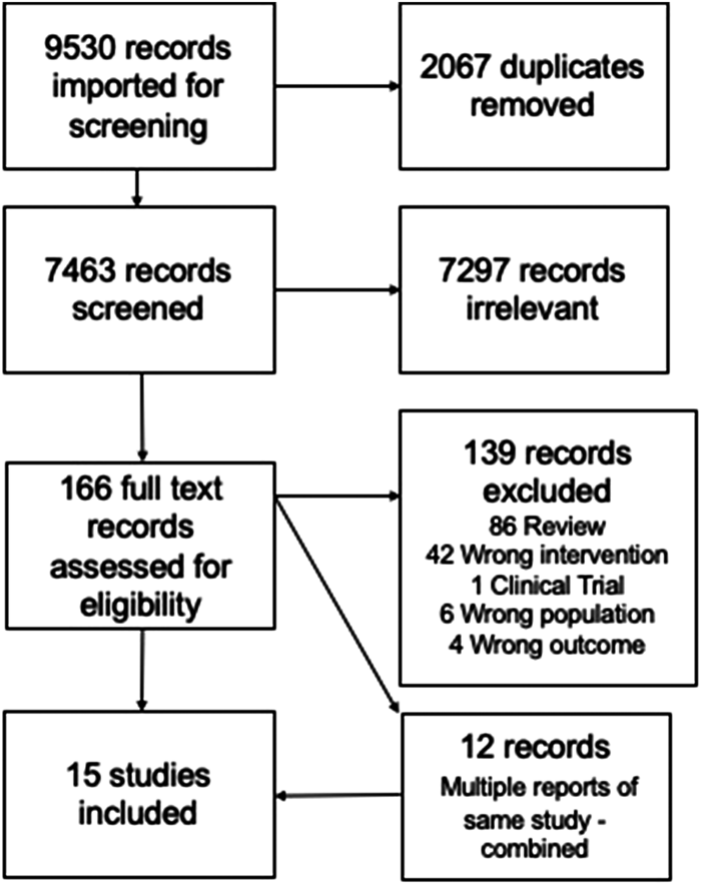
Preferred Reporting Items for Systematic Review and Meta‐Analysis (PRISMA) flow diagram of study selection.

After the search, 9530 reports were imported for screening (CINAHL = 1333, Cochrane = 442, EMBASE = 5119, Medline = 2272, PsycInfo = 359, Grey Literature = 5). Figure 1 illustrates the study selection process. Reports that detailed the same study were collated into one study. Fifteen studies were included in the final analysis. Some studies reported assessment of resources they described as transplant decision aids but were excluded for failing to meet the IPDAS criteria for being a PDA. As per the IPDAS criteria, a key component that distinguishes a PDA is it “helps patients to recognize that the decision is value sensitive and to clarify the values they place on the harms and benefits.^50^” The “My Kidney, My choice decision aid” by Fortnum et al. was excluded because it did not detail the risks/benefits of transplantation nor encourage the value clarification around transplantation.^72^ Weng et al., Barnieh et al., and Reif Bergman et al. were excluded as the PDAs did not elicit value clarification.^73^, ^74^, ^75^ Lee et al. described their intervention as a PDA but gave insufficient information about the intervention and did not respond to requests for further material.^76^

### 3.2 Study characteristics

Of the final 15 studies, there were 4278 participants (Table 1). Eight studies were RCTs, five were pre‐test/post‐test studies, and two were non‐randomised studies (Prieto‐Velasco et al. was a prospective registry study comparing the outcomes of those who used PDAs to standard education with no randomised arm; Mucsi et al. undertook a non‐randomised parallel arm control study). Most PDAs were focused on kidney transplant‐related decisions (*n* = 12). The demographics of participants are in Table S6. There was a spread of educational levels indicating generalizable results. Ethnicity was skewed by several studies which exclusively focused on populations with black race. The most common decision assessed was whether to have a transplant; however, two PDAs assessed whether to accept a transplant from an increased viral risk donor and two PDAs assessed whether to accept a higher prognosis risk organ.

**TABLE 1:** Characteristics of all included studies.

Study identifier	PDA name	Decision	Study population	Organ	Location	Enrolment	* n*	Study design	Outcomes
Axelrod (2017)^32^	My Transplant Coach	Kidney transplant versus dialysis	Potential recipient	Kidney	USA	2015—2016	81	Pre‐test Post‐test	K, A, I, AE
Boulware (2018)^34^	PREPARED	Kidney transplant versus dialysis	Potential recipient	Kidney	USA	2012–2013	92	RCT	B, A
Dubin (2019)^35^	Modality Decision Program	Kidney transplant versus dialysis	Potential recipient, clinician	Kidney	USA	NR	28	Pre‐test Post‐test	K, SE, CM
Gordon (2017)^36^	Inform Me	Increased viral risk donor versus standard viral risk	Potential recipient	Kidney	USA	2013–2014	288	RCT	K, CM
Kayler (2020)^37^	Simplify KDPI IRD‐1‐2‐3	Low versus high KDPI kidney and increased viral risk donor versus standard viral risk donor	Potential recipient, carers	Kidney	USA	2019	144 (80 recipients, 64 care givers)	RCT	K, SE, CM, B, A
Mucsi (2018)^47,^ ^33^	Explore Transplant Ontario	Kidney transplant versus dialysis	Potential recipients	Kidney	Canada	2016–2017	230	Prospective cohort	K, R
Patzer (2018)^38^	iChooseKidney	Kidney transplant versus dialysis	Potential recipients, clinicians	Kidney	USA	2014–2015	443	RCT	K, DC, CM, B, RP, A, C, AE
Polo (2020)^39^	Informed Choices Cystic Fibrosis Decision Aid	Lung transplant versus conservative care	Potential recipient, carers, clinicians	Lung	USA	NR	42	Pre‐test Post‐test	K, DC, A
Prichard (2013)^40^	Chronic Kidney Disease: Option Grid	Kidney transplant versus dialysis	Potential recipients	Kidney	UK	NR	65	Pre‐test Post‐test	K, SE
Prieto‐Velasco (2015)^41^	Education Process	Kidney transplant versus dialysis	Potential recipients	Kidney	Spain	2010—2012	1044	Prospective cohort	CM, B
Vandemheen (2009)^42^	Lung Transplant Decision aid for people with Cystic Fibrosis	Lung transplant versus conservative care	Potential recipients	Lung	Canada Australia	2006–2008	149	RCT	K, DC, CM, RP, A, VC,DD, SE
Volk (2014)^43^	Liver Quality Decision Aid	Livers with different graft survival	Potential recipients	Liver	USA	NR	56	Pre‐test Post‐test	K, CM, SE, RP, C,
Waterman (2018)^44^	Explore Transplant	Kidney transplant versus dialysis	Potential recipients	Kidney	USA	2007–2008	253	RCT	K, SE, R, I,B
Waterman (2019)^46^	Explore Transplant at Home	Kidney transplant versus dialysis	Potential recipients	Kidney	USA	2014–2016	561	RCT	K, SE, I, B,
Waterman (2020)^45^	Your Path to Transplantation	Kidney transplant versus dialysis	Potential recipients	Kidney	USA	2014–2017	802	RCT	K, R, B, A

*Note*: The study populations document and demographics described are referring to patients included in the studies.

Abbreviations: A, Acceptable; AE, Adverse effects; B, Behavior; C, communication; CM, Choice Made; DC, Decisional conflict; DD, durability of decision; I, Feeling informed; IRD, Increased Viral Risk Donor; K, knowledge; KDPI, Kidney Donor Profile Index; NR, Not Reported; R, Readiness; RCT, Randomized Controlled Trial; RP, Risk Perception; SE, self efficacy; UK, United Kingdom; USA, United states of America; VC, Value Congruence.

### 3.3 Risk‐of‐bias assessment

**FIGURE 2:**
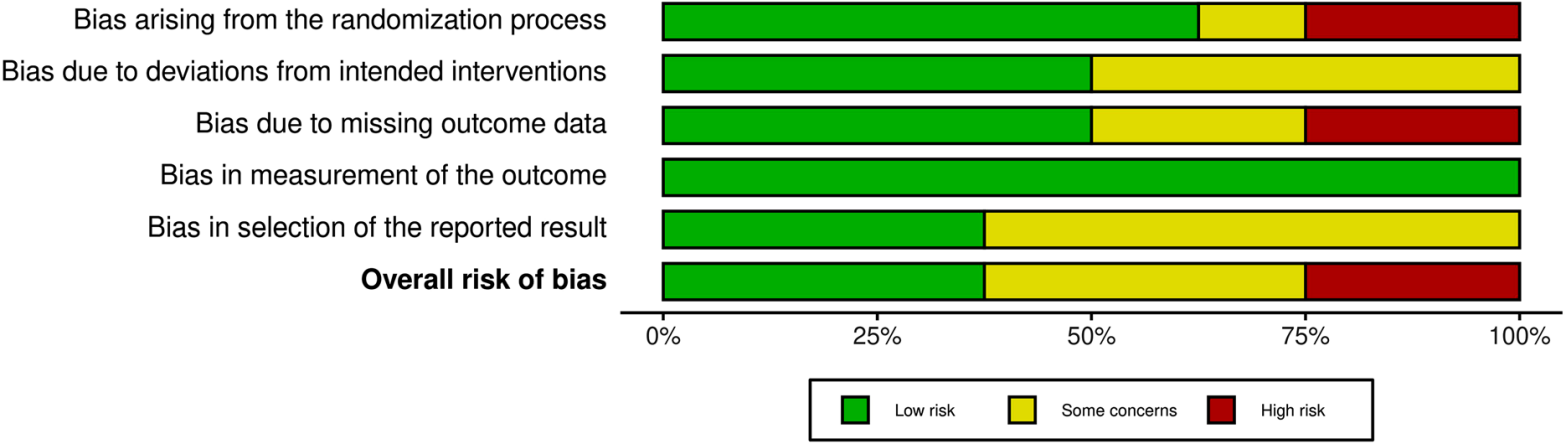
Summary plot of risk‐of‐bias domains for all eight (53%) randomized controlled trial (RCT) studies in the review (by domains).

Risk‐of‐bias was assessed for knowledge. When knowledge was not included, then the primary outcome of the study was used to assess risk‐of‐bias including actions to pursue transplant (behavior)^34^ and choice made.^41^ Figures 2 and S1–S4 show the risk‐of‐bias assessment. The risk for the RCTs varied from low to high (Figures 2 and S1). Two studies had problematic randomisation processes and missing outcome data leading to high risk‐of‐bias. Pritchard et al.^40^ had a high risk‐of‐bias due to the pragmatic study design (staff determining study enrolment were influenced by time limitations). Two studies were abstracts^33^, ^39^, ^47^ so the details required for risk‐of‐bias assessments were limited by word count. Prieto‐Velasco et al. had a critical risk‐of‐bias due to confounding (all patients were offered the PDA and those who declined were used as the comparison group).

### 3.4 Interventions

There were a variety of formats and modes of delivery used for the PDAs (Table S7). There were also variable environments for PDA usage; some were used by patients alone while others were used within consultations. All the PDAs have been described using the SUNDAE checklist (Table S8).

### 3.5 Outcomes

Several outcomes were assessed in the included studies (Table 1). These are listed below and described in greater detail within the Supplementary Methods.

#### 3.5.1 Knowledge

**FIGURE 3:**
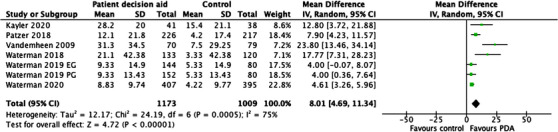
Forest plot of mean knowledge difference (before and after the intervention) for randomized control trials for PDA compared to controls (standard of care). Mean knowledge scores and standard deviations have been scaled to be out of 100 to allow comparison. Waterman et al. (2019)^46^ had the control arm split due to comparison of two interventions. EG, Educator Guide; PDA, Patient Decision Aid; PG, Patient Guided.

Eighty seven percent of studies (*n* = 13) assessed knowledge. All demonstrated an increase in knowledge with PDA use. Seven RCTs assessed knowledge (Table 2) and six could be combined for meta‐analysis (Figure 3). This favored PDA to control with a mean knowledge difference of 8.01 on the 0–100 scale (95% CI 4.69–11.34, *p* < .00001).

**TABLE 2:** Outcome of knowledge for RCT, non randomized studies and pre‐test post‐test studies.

Study identifier	Scale	Timing of assessment	PDA (*n*)	Control (*n*)	Knowledge change PDA	Knowledge change control	Notes
Randomized control trial							
Gordon (2017)	31 MCQ	During and 1 week	133	155	NA	NA	Post‐test only design
Kayler (2020)	Nine item knowledge scale	Immediately after	41^b^	38^b^	2.54 (1.8)	1.39 (1.9)	*p* = .009
Patzer (2018)	Nine item knowledge scale	Immediately after	225	217	1.09 (2.0)	.38 (1.8)	*p* < .0001
Vandemheen (2009)	Four MCQ	3 weeks	70	79	1.24 (1.38)	.3 (1.17)	*p* < .0001
Waterman (2018)^44^	Nine true/false, nine MCQ	1 month	133	120	3.8	.6	*p* < .001
Waterman (2019)^46^	15 item scale	8 months	152	160	1.4	.8	*p* = .01
Waterman (2020)^45^	11 true/false, eight MCQ	8 months	407	395	4.46	2.13	Means scaled to out of 100. *p* < .001
Non randomized study							
Mucsi (2018)	19 item score	6 months	124	106	1.92 (2.7)	.79 (2.7)	*p* = .01
Pre‐test Post‐test study							

Study ID	Scale	Timing of assessment	Before mean (*n)*	After mean (*n*)	Scaled mean change %		Statistical significance
Axelrod (2017)	20 score	Shortly after	9.1 (81)	13.8 (81)	13		*p* < .001
Prichard (2013)	Six item score	2 months	67 (65)	84 (39)	17		NR
Dubin (2019)	18 MCQ	1 month	65 SD 56 (25)	83 SD14 (25)	18		*p* < .001
Volk (2014)	Two questions	Immediate	56.5 (53)	97 (53)	41		*p* < .001
Polo (2020)	NR	1 month	NR (21)	NR (21)	.85^a^		*p* = .0297 *^b^mean change raw values not reported*

*Note*: For RCT the knowledge before and after use of intervention is reported for PDA compared to controls. This is the same for non‐randomized studies however as they cannot be compared they are reported separately. For pre‐test/post‐test studies the change in knowledge is reported for the whole cohort before and after intervention without a comparison arm.

Abbreviations: MCQ, Multiple Choice Question Scores; NR, Not Reported; PDA, Patient Decision Aid; RCT, Randomized Controlled Trial; SD, Standard Deviation.

^a^ Derived from graph.

^b^ The sample size refers to patients who used the PDA. ^b^mean change raw values not reported.

##### Heterogeneity

There was moderate statistical heterogeneity with an I^2^ value of 75%, however, most of the CIs overlap.

##### Sensitivity analysis

There was no change in the direction or strength of effects for the meta‐analysis with subgroup‐analysis restricted to kidney transplants or excluding high‐risk‐of‐bias studies. There was no change when removing PDAs which looked at the complexities of decisions about transplant (i.e., high viral risk donors), rather than whether to have a transplant or not.

##### Reporting bias

A funnel plot (Figure S5) suggests no publication bias.

##### Certainty of evidence

For knowledge for the RCTs the certainty of evidence was low. This was down‐graded from high due to risk‐of‐bias and inconsistency based on the moderate heterogeneity from the I^2^ value as per the GRADE methodology by Cochrane. There was one non‐randomized trial design that had low certainty evidence. The pre‐test/post‐test studies (Table 2) also showed a statistically significant increase in knowledge though there is no comparison for this outcome.

#### 3.5.2 Accuracy of risk perception

Two studies found strong evidence that the PDA improved risk perception.^42^, ^43^ This is defined as whether patients could accurately judge the probability of an outcome for an individual with similar characteristics to themselves.

#### 3.5.3 Acceptability

The acceptability of the PDA (whether it helped users make a decision) was examined in nine studies.^32^, ^34^, ^35^, ^36^, ^37^, ^38^, ^39^, ^42^, ^45^ Overall, 83% of patients found the PDA helped them to decide (Table S9). 85%–100% of patients would recommend the PDA to someone else.^35^, ^37^, ^42^ All three studies including clinician participants found that the majority considered the PDA acceptable.^35^, ^38^, ^39^ Patzer et al. found that 95% (18/19) of clinicians thought they could benefit from the PDA implementation. Dubin et al. found 95% (21/22) of clinicians thought the PDA helped patients prepare for kidney failure, and 95% felt the PDA helped them understand patients’ values and preferences. Kayler et al. also examined acceptability in carers who mostly felt that the PDA was acceptable.

#### 3.5.4 Adverse effects

Any reported adverse outcomes were included. Patzer et al. noted that PDA use increased appointment length. Axelrod et al. identified that 17% (*n* = 14) of users found the survival graphs upsetting to view. There were no other reported adverse outcomes for cost or health impacts.

#### 3.5.5 Behavior

Forty seven percent (*n* = 7) of studies assessed behavior outcomes, defined as whether the intervention led to any behaviour change.^34^, ^37^, ^38^, ^41^, ^44^, ^45^, ^46^ Several studies assessed choice by assessing steps pursuing transplantation. Waterman et al. (2018)^44^ found participants using PDAs had greater odds of taking actions to pursue transplant than the control group. Waterman et al. (2019)^46^ found evidence of more steps in the patient‐guided PDA group (incident rate ratio [IRR]: 1.21, 95% CI: 1.01–1.47, *p* = .04). Waterman et al. (2020)^45^ also found evidence of a difference in steps in the PDA group compared to control (relative risk: 1.12, 95% CI: 1.01–1.24, *p* = .034). Two studies found no difference in steps between intervention and control.^34^, ^38^

Kayler et al. assessed choice by how many patients signed a consent to receive Kidney Donor Profile Index (KDPI) offers > 85% (these are considered offers of kidneys with a worse prognosis) and found there were marginally more in the PDA than control (PDA 27.5%, control 13.5%, *p* = .13).

Three studies assessed choice through health outcomes. Waterman et al. (2018)^44^ found more live donors presented for kidney transplant evaluation for the PDA group compared to control after 2 years (IRR: 2.05, 95% CI: 1.00–4.31, *p* = .05). There was no evidence of a different rate of transplantation at 1 year. There was weak evidence of a difference at 3‐years, with higher transplant rates in the PDA compared to control (PDA 10.8%, control 5.2%, *p* = .09). Waterman et al. (2020)^45^ also found PDA users were more likely to have received a living donor transplant or be waitlisted for deceased donor transplantation compared to control after 18‐month (HR: 1.39, 95% CI: 1.12–1.74, *p* = .003). Prieto‐Velasco et al. found low rates of pre‐emptive living‐donor kidney transplants in both groups (1% PDA,0% control).

#### 3.5.6 Choice made

Two studies assessed whether patients made a choice after PDA use. Prieto‐Velasco et al. found 58% made a choice after PDA use but had no unbiased comparison arm. Dubin et al. found that the proportion who made a choice increased from 32% to 100% post PDA.

Several studies assessed which choice patients made after using the PDA. Prieto‐Velasco et al. found 3.2% of patients chose pre‐emptive living donor transplantation. Dubin et al. found that 48% (12/25) chose transplantation at baseline which increased to 84% (21/25, *p* = .01) after using the PDA. Patzer et al. found the proportion who changed their decision was similar between control and PDA groups. Vandeheem et al. found a similar proportion chose transplant in control and PDA groups (Before: 50% PDA, 53% control. After: 67% PDA, 70% control).

Three studies assessed the patient's choices regarding increased viral risk donors after PDA use.^36^, ^37^, ^43^ Gordon et al. found no change in willingness to accept an increased viral risk kidney after PDA use (mean difference .28, 95% CI .61–.04, *p* = .09). Kayler et al. found greater willingness to accept an increased viral risk offer after PDA compared to control (Beta‐coefficient .07, 95%CI .25–1.16, *p* = .03).

Two studies examined choices about higher prognosis risk grafts.^37^, ^43^ Kayler et al. found there was no difference in acceptance of higher KDPI kidney transplants when comparing PDA to control groups. Volk et al. found patients had higher mean willingness scores when considering a high KDPI organ after PDA use (difference before: 3.53, after:4.6, *p* < .001).

#### 3.5.7 Communication

Two studies examined patient‐clinician communication.^38^, ^43^ Patzer et al. found the proportion of clinicians who discussed survival‐benefit was higher with the PDA compared to controls: kidney transplant survival benefit (PDA 95% vs. Control 90%, *p* = .04), benefits of living compared to deceased donor transplantation (PDA 91% vs. Control 78%, *p* < .001), and benefit of transplantation compared to dialysis (PDA 97% vs. Control 94%, *p* = .08). Volk et al. found patients felt no change in confidence when talking to clinicians after using the PDA.

#### 3.5.8 Decisional conflict

Three studies reported on decisional conflict, a standardized measure of uncertainty around a decision.^38^, ^39^, ^42^ Vandeheem et al.^42^ showed lower decisional conflict score in the PDA group compared to control (PDA mean 11.6, SD13.6 vs. control 20.4, SD16.9, *p* = .0007). Patzer et al.^38^ showed no difference between the control and PDA groups. Polo et al. showed a reduction in decisional conflict after PDA use (mean reduction 10.86, *p* = .05).

#### 3.5.9 Durability of decision

Vanderheem et al. found no difference in the durability of choice (proportion of participants maintaining their initial decision 1 year later) between PDA and control.^42^

#### 3.5.10 Feeling informed

Three studies assessed the proportion of patients who felt informed after using the PDA.^32^, ^44^, ^46^ All studies showed a statistically significant difference, with PDAs being associated with a greater proportion of patients feeling informed compared either to before use or to comparator (Table S10).

#### 3.5.11 Readiness

Two studies assessed decision readiness based on a validated model measuring stages of change.^44^, ^45^ Waterman et al. (2018)^44^ showed increased readiness to be on the deceased donor waiting‐list (OR 2.34, 95%CI 1.2–4.57, *p* = .01). Waterman et al. (2020)^45^ showed increased readiness to receive a deceased donor kidney transplant (OR 3.16, 95%CI .92–5.39, *p* = .019) and increased readiness to pursue living donor transplantation (OR 3.77, 95% CI 1.04–6.50, *p* = .005).

#### 3.5.12 Self‐efficacy

Seven studies assessed self‐efficacy with patients grading their ability to make decisions.^35^, ^37^, ^40^, ^42^, ^43^, ^44^, ^46^ Four studies showed no difference, and three studies showed an improvement. Dubin et al. in their pre‐test/post‐test study^35^ showed an improvement in decision efficacy (baseline mean 3.7, SD0.7, 1‐month 4.3, SD0.5, *p* < .001). Vandeheem et al. showed a difference between PDA and control groups after the interventions (mean scores; PDA, 65.1, SD24.9, Control 53.8, SD27.1, *p* = .009). Prichard et al. reported an increase in self efficacy from 52% to 80% but undertook no statistical analysis.

#### 3.5.13 Value choice congruence

One study assessed value choice congruence^42^ which is whether the patients’ values aligned with the choice they made and found patient choices were in line with values in both PDA and control groups.

## 4 DISCUSSION

This review supports the use of PDAs to increase knowledge compared to standard education for solid organ transplantation. PDAs are mostly considered an acceptable tool by clinicians and consumers, however, it is difficult to determine whether they promote shared decision‐making due to the lack of information about non‐knowledge‐based outcomes of PDA use. There was some evidence that PDAs may improve accuracy of risk perception, proportion of patients feeling informed and readiness to decide, however, the evidence of an effect was weak. The impact of PDAs on behavior change and choice made was examined in few studies and the direction of effect was inconsistent. Several other outcomes were examined but conclusions are limited by the outcome measurements’ heterogeneity and infrequent assessment.

Our study is the first meta‐analysis to demonstrate that organ transplantation PDAs increase knowledge though the effect size is small. The results of this study align with previous systematic reviews on PDAs in other fields. However, while knowledge was the most‐frequently assessed outcome in this review, this is only one component of decision quality. The main differentiating feature of a PDA from educational material is the focus on eliciting values and aligning these with the different treatment options. Therefore, assessing value congruence should be a core outcome. Unfortunately, this has not been adequately examined in solid organ transplantation PDA trials to date and so we are unable to conclude the impact of PDAs on other markers of decision quality in this review. Additionally, to understand the utility of PDAs to facilitate shared decision‐making in transplantation, we also need to assess whether these tools increase patient involvement in their decisions; this was not investigated in the included studies. This is particularly important given the time and cost required to develop a PDA.

There are important strengths to this study. We had broad inclusion criteria and undertook a comprehensive literature appraisal. Using a rigorous systematic approach, we have provided evidence for transplantation PDA to improve knowledge transfer. The study samples incorporated several race/ethnicity groups including black, Hispanic, white, and other which may support PDA use as effective and acceptable within a diverse population, however, further study would be beneficial.

There are also weaknesses of the review. This review identified a range of outcomes, using many different outcome measures. For future research, it would be constructive to have unifying validated outcome measures. This aligns with findings from previous work on PDAs in other areas.^77^ Additionally, several of the studies were not methodologically robust. Both these factors limit confidence in our estimates of effects. No studies examined patient participation in decision‐making and so it is unclear whether PDAs improve shared decision‐making. Participants may be more likely to support PDAs a priori, contributing to selection bias and limiting generalizability. Additionally, to increase the comprehensiveness of our review, we assessed all organ transplants, however, arguably decisions about different organ transplants are too different to be grouped. Similarly, the decision about accepting a high viral risk donor organ is different from choosing to have a transplant. Given this is a developing field with limited studies, we felt it was helpful to assess all PDAs together, however, with greater numbers of studies, these individual decisions could be examined in isolation in the future.

## 5 CONCLUSION

In conclusion, our review demonstrates that PDAs increase knowledge and are mostly considered acceptable with few adverse outcomes when making decisions about solid organ transplantation. These results support the ongoing use and development of these tools as they have potential to improve the transplant‐related knowledge of patients with organ failure. Further work, however, is needed on the impact of PDAs on other markers of decision‐making.

## AUTHOR CONTRIBUTIONS

Georgina L. Irish: Substantially contributed to the design of the work, undertook screening, data extraction, risk‐of‐bias assessment, analysis of the data, drafting of the manuscript, and gave final approval. Alison Weightman: Undertook screening, data extraction, the risk‐of‐bias assessment, participated in critical revision of the paper and gave final approval. Jolyn Hersch: Resolved conflicts as a third reviewer, contributed to analysis and interpretation of the data, participated in critical revision of the paper, and gave final approval. P. Toby Coates: Substantially contributed to the interpretation of data for the work, participated in critical revision of the paper, and gave final approval. Philip A Clayton: Substantially contributed to the conception of the work, resolved any conflicts as a third reviewer, contributed to analysis and interpretation of the data, participated in critical revision of the paper, and gave final approval.

## CONFLICT OF INTEREST

The authors declare no conflicts of interest.

## Supporting information

Supplementary Information

## Data Availability

Data sharing is not applicable to this article as no new data were created or analyzed in this study.
