# Supplementary material for: Do patient decision aids help people who are facing decisions about solid organ transplantation? A systematic review
Source: Clin Transplant. 2023 Feb 19;37(4):e14928. doi: 10.1111/ctr.14928 (PMC10909430; doi:10.1111/ctr.14928)
Supplement: Supplementary file 1 — Supplementary Information [file CTR-37-e14928-s001.docx]

**Supplementary Appendix**

**Contents**

Supplementary Methods

Supplementary Tables

# Table S1: Search strategy for Medline

# Table S2: Search strategy for Embase

Table S3: Search strategy for Psych Info

Table S4: Search strategy for CINAHL

Table S5: Search strategy for Cochrane

Table S6: Table detailing demographics of participants in the studies.

Table S7: table describing format and administration of PDA.

Table S8: SUNDAE checklist table.

Table S9: Proportions of patients who felt PDA was acceptable

Table S10: Proportion of patients who reported feeling informed to make a decision about LD or DD kidney transplantation.

Supplementary Figures

Figure S1: Traffic light plot for Risk of Bias 2^62^ domains for all RCT studies included in the review for the outcome of knowledge (study by study). Boulware et. al did not assess knowledge, and therefore risk of bias was based on the primary outcome which was actions to pursue transplant. ^67^

Figure S2: Summary plot of National Institutes of Health risk of bias domains for pre-test post-test studies (by domains).

Figure S3: Traffic light plot National Institutes of Health risk of bias domains for all pre-test post-test studies (study by study).

Figure S4: Traffic light plot for non-randomised studies using ROBINS I^63^ risk of bias tool

Figure S5: Forest plot assessing for reporting bias for the outcome of knowledge for all RCT included in the meta-analysis.

**Supplementary methods**

Outcome assessment:

For comprehensiveness, all outcomes in any study included were assessed as part of this systematic review. The outcomes reported were acceptability, accuracy of risk perception, adverse effects, behaviour, choice made, communication, decisional conflict, durability of a decision, feeling informed, readiness, self-efficacy, and value congruence. The definition and methods for synthesis are described below:

*Accuracy of risk perception*

Two studies (13%) assessed the effect of PDA on risk perception^42,43^. This is defined as whether patients could accurately judge the probability of an outcome for an individual with similar characteristics to themselves.

*Acceptability*

Whether the PDA helped to make a decision was examined in 9 studies (60%).^32,34-39,42,45^ 6 studies assessed acceptability to patients and 3 studies assessed acceptability for clinicians. Whether a PDA was considered helpful was defined different by the different studies and self-reported by patients. This was collected as the proportion of the cohort who felt the PDA was helpful and presented as a percentage. One study compared this to a control group. Meta-analysis was not feasible as only one study had a control. The outcome was tabulated for synthesis and overall proportion who felt the PDA was acceptable was averaged.

*Adverse effects*

2 studies examined adverse outcomes from PDA in the form of feedback. Any reported adverse outcomes reported on was listed by the studies. These were collated and described for this review.

*Behaviour*

47% (n=7) of studies assessed behaviour outcomes, such as whether patients followed through on their choice to pursue transplantation as an option.^34,37,38,41,44-46^ Behaviour as an outcome, was whether the intervention led to any behaviour change. These included steps to pursue transplantation and the outcome of transplantation itself. These outcomes had a variety of definitions: 2 assessed whether patients took steps to transplantation, 1 looked at whether patients signed consent, 2 looked at rates of transplantation, 1 assessed the rates of whether of patient presented for evaluation. The many different definitions of how behaviour was defined meant and how these were analysed mean that synthesis of this outocme was descriptive.

*Choice: Choice made*

47% (n=7) of studies assessed choice made or preferred choice as a surrogate for choice behaviour.^35-38,41-43^ The outcome of choice after using a PDA was assessed in different ways. Two studies assessed whether patients made a choice at all after using the PDA. The other studies about choice focused on which choice was made. For 3 studies this deicison was whether to have a solid organ transplant. For 3 studies the decision assessed was whether a patient would accept a donor of high viral risk. For 2 studies, the deicison was whether to accept an organ from a donor with higher prognosis risk. Given the small number of studies which collected this outcome and the heterogeneity in the definition of a choice, these outcomes were descriptively synthesied.

*Communication*

Two studies examined patient-clinician communication.^38,43^ Patzer et al. assessed this as the proportion of clinicians who discussed survival benefit with higher with the PDA compared to controls. Volk et al. assessed this by whether patients felt any change in confidence when talking to clinicians after using the PDA.

*Decisional conflict*

Three studies reported on decisional conflict.^38,39,42^ A decisional conflict scale measures the feeling of uncertainty around a decision. Two studies used the O’Connor decisional conflict scale which is scored from 0-100, where 0 means low decisional conflict and 100 is high decisional conflict.^78^

*Durability of decision*

One study examined durability of the decision when assessed at 12 months.^42^ The was defined as whether the decision was the same after 12 month as a proportion.

*Feeling informed*

3 studies assessed the proportion of patients who felt informed after using the PDA. 3 studies collected the outcome of whether a patient felt informed after using a PDA compared to control. This was presented as a proportion who felt informed as a percentage. One study who assessed this was a pre-test post-test study which compared portion who felt informed before and after PDA use. 2 studies were RCT which compared proportion informed after PDA to proportion informed after control use. These data were stratified by whether the patient felt informed about living or deceased donation. These data were too small to allow synthesis so are presented in tabulated form.

*Readiness*

Two studies assessed patient readiness to make a decision.^44,45^ This was based on a validated model measuring stages of change.^79,80^

*Self-efficacy*

47% of studies assessed self-efficacy (n=7)^35,37,42-44,46^ with patients grading their ability to make decisions. Four studies showed no difference, and three studies showed an improvement. Dubin et al. in their pre-test/post-test study^35^ showed an improvement (baseline mean 3.7 SD 0.7, 1-month mean 4.3 SD 0.5, p<0.001). Vandeheem et al. showed a difference between PDA and control groups after the interventions (mean scores; PDA 65.1,SD 24.9, Control 53.8,SD 27.1, p=0.009). Prichard et al. showed an increase in self efficacy from 52% to 80% but undertook no statistical analysis.

*Value choice congruence*

One study assessed value choice congruence which is whether the patients’ values aligned with the choice they made. This was defined by the study authors, for example if a potential lung transplant recipient chose lung transplantation as the preferred choice, then a value congruent choice would be that they considered living longer as very important and placed less importance on the stress and invasiveness of lung transplantation.

***Supplementary Tables***

*Search strategy*

*Medline*

| Population | Intervention |
| --- | --- |
| exp *Organ Transplantation OR  exp *Tissue Donors  OR  exp *Transplant Recipients  OR  Organ Transplant*.tw  OR  Kidney Transplant*.tw  OR  Renal Transplant*.tw  OR  Lung Transplant*.tw  OR  Liver Transplant*.tw  OR  Hepatic Transplant*.tw  OR  Heart Transplant*.tw  OR Heart-lung Transplant*.tw  OR  Cardiac Transplant*.tw  OR  Pancrea* Transplant*.tw | Exp *Decision Making, Shared  OR  exp *Decision Support Techniques  OR  exp *Decision Making  OR  decision making.tw  OR  Decision Aid*.tw  OR  Decision Tool*.tw  OR  Decision Application*.tw  OR  Decision Instrument*.tw  OR  Decision Technolog*.tw  OR  Decision method*.tw  OR  Decision intervention*.tw  OR  Decision material*.tw  OR  Decision process*.tw  OR  Decision program*.tw  OR  Decision technique*.tw |

# *Table S1: Search strategy for Medline*

# *Embase*

| Population | Intervention |
| --- | --- |
| “Organ Transplantation”/expOR“Organ Donors”/expOR“Graft Recipients”/expOR“Organ Transplant*”:ti,abOR“Kidney Transplant*”:ti,abOR“Lung Transplant*”:ti,abOR“Liver Transplant*”:ti,abOR“Hepatic Transplant*”:ti,abOR“Heart Transplant*”:ti,abOR“Pancreas Transplant*”:ti,abOR“Renal Transplant*”:ti,abOR“Cardiac Transplant*”:ti,abOR Heart-Lung Transplant*:ti,ab | “decision support system”/expOR“decision making”/expOR“decision making”:ti,abOR “decision aid*”:ti,ab  OR  “decision making*”:ti,ab  OR  “decision support*”:ti,ab  OR  “Decision tool*”:ti,ab  OR  “Decision instrument*”:ti,ab  OR  “Decision technolog*”:ti,ab  OR  “Decision technique”:ti,ab  OR  “Decision system*”:ti,ab  OR  “Decision program*”:ti,ab  OR  “Decision process*”:ti,ab  OR  “Decision method*”:ti,ab  OR  “Decision Intervention*”:ti,ab  OR  “Decision material*”:ti,ab |

# *Table S2: Search strategy for Embase*

# *Psych Info*

| Population | Intervention |
| --- | --- |
| Exp Organ TransplantationORExp Tissue DonationOROrgan Transplant.ti,abORKidney Transplant.ti,abORLung Transplant.ti,abORLiver Transplant.ti,abORHeart Transplant.ti,abORPancreas Transplant.ti,abORRenal Transplant.ti,abORCardiac Transplant.ti,abORHeart-Lung Transplant.ti,ab | Exp decision support systemORExp decision makingOR decision aid.ti,ab  OR  decision making.ti,ab  OR  decision support.ti,ab  OR  Decision tool.ti,ab  OR  Decision instrument.ti,ab  OR  Decision technology.ti,ab  OR  Decision technique.ti,ab  OR  Decision system.ti,ab  OR  Decision program.ti,ab  OR  Decision process.ti,ab  OR  Decision method.ti,ab  OR  Decision Intervention.ti,ab  OR Decision material.ti,ab |

# *Table S3: Search strategy for Psych Info*

*CINAHL with full text*

| Population | Intervention |
| --- | --- |
| MH”Organ Transplantation+”ORMH”Transplant Recipeint”ORMH“Transplant Donors”ORTI”Organ Transplant”ORTI”Kidney Transplant”ORTI”Lung Transplant”ORTI”Liver Transplant”ORTI”Heart Transplant”ORTI”Pancreas Transplant”ORTI”Renal Transplant”ORTI”Cardiac Transplant”ORTI”Heart-Lung Transplant”ORAB”Organ Transplant”ORAB”Kidney Transplant”ORAB”Lung Transplant”ORAB”Liver Transplant”ORAB”Heart Transplant”ORAB”Pancreas Transplant”ORAB”Renal Transplant”ORAB”Cardiac Transplant”ORAB”Heart-Lung Transplant” | MH“Decision Support Systems, Clinical+”ORMH “decision making+”OR TI“decision aid” OR AB“decision aid”  OR  TI“decision making”  OR  TI“decision support”  OR  TI“Decision tool”  OR  TI“Decision instrument”  OR  TI“Decision technology”  OR  TI“Decision technique”  OR  TI“Decision system”  OR  TI“Decision program”  OR  TI“Decision process”  OR  TI“Decision method”  OR  TI“Decision Intervention”  Or\R  TI“Decision material”  OR  AB“decision making”  OR  AB“decision support”  OR  AB“Decision tool”  OR  AB“Decision instrument”  OR  AB“Decision technology”  OR  AB“Decision technique”  OR  AB“Decision system”  OR  AB“Decision program”  OR  AB“Decision process”  OR  AB“Decision method”  OR  AB“Decision Intervention”  OR  AB“Decision material” |

# *Table S4: Search strategy for CINALH*

| Population | Intervention |
| --- | --- |
| MeSH descriptor: [Decision Support Techniques] explode all trees  OR  MeSH descriptor: [Decision Making] explode all trees  OR  ((decision aid) OR (decision making) OR (decision support) OR (Decision tool) OR (Decision instrument) OR (Decision technology) OR (Decision technique) OR (Decision system) OR (Decision program) OR (Decision process) OR (Decision method) OR (Decision Intervention) OR (Decision material)):ti,ab,kw (Word variations have been searched) | MeSH descriptor: [Transplants] explode all trees  OR  MeSH descriptor: [Transplant Recipients] explode all trees  OR  MeSH descriptor: [Tissue and Organ Procurement] explode all trees  OR  ((Organ Transplant) OR (Kidney Transplant) OR (Lung Transplant) OR (Liver Transplant) OR (Heart Transplant) OR (Pancreas Transplant) OR (Renal Transplant) OR (Cardiac Transplant) Or (Heart-Lung Transplant).):ti,ab,kw (Word variations have been searched) |

# *Table S5: Search strategy for Cochrane*

*Supplementary results*

| **Study Identifier** | **Intervention** | **Male sex, %** | **Age** | | **Ethnicity/Race** | | | | **Education** | | | |
| --- | --- | --- | --- | --- | --- | --- | --- | --- | --- | --- | --- | --- |
|  |  |  | Mean, SD | Median, IQR | Black % | Hispanic % | Other % | White % | 8th grade or less % | High school (^+^ or less) % | Undergraduate school % | Graduate school % |
| **Axelrod 2017** | PDA | 51 | 52, 14 |  | 26 | 15 | 32 | 27 | NR | 20^+^ | 55 | 23 |
| **Boulware 2018** | PDA | 50 | 53, 16 |  | 100 | 0 | 0 | 0 | NR | 77^+^ | 14 | 10 |
| **Boulware 2018** | PDA + financial assistance program | 39 | 55, 13 |  | 100 | 0 | 0 | 0 | NR | 74^+^ | 20 | 7 |
| **Boulware 2018** | Control | 58 | 52, 12 |  | 100 | 0 | 0 | 0 | NR | 68^+^ | 32 | 0 |
| **Dubin 2019** | PDA | 68 | 65, 15 |  | 20 | NR | 32 | 48 | NR | 16^+^ | 40 | 28 |
| **Gordon 2017** | PDA | 58.6 | 51.2, 11.3 |  | 55 | NR | 14 | 32 | NR | 73^+^ | 27 | NR |
| **Gordon 2017** | Control | 62.6 | 50.5, 12.3 |  | 55 | NR | 12 | 33 | NR | 72^+^ | 28 | NR |
| **Kayler 2020** | PDA KDPI | 66.7 | 61.1, 10.4 |  | 23 | NR | 10 | 67 | NR | 62^+^ | 39 | NR |
| **Kayler 2020** | Control | 69.4 | 58.7, 10.8 |  | 33 | NR | 11 | 56 | NR | 53 | 47 | NR |
| **Kayler 2020** | PDA IRD | 66.7 | 61.1, 10.4 |  | 23 | NR | 11 | 67 | NR | 62^+^ | 39 | NR |
| **Mucsi 2018** | PDA | NR | 63, 10 |  | NR | NR | NR | NR | NR | NR | NR | NR |
| **Mucsi 2018** | Control | NR | 55, 14 |  | NR | NR | NR | NR | NR | NR | NR | NR |
| **Patzer 2018** | PDA | 63.3 | 51.1, 9.9 |  | 50 | 11 | 7 | 32 | 2 | 29 | 53 | 14 |
| **Patzer 2018** | Control | 61.8 | 50.1, 10.3 |  | 45 | 10 | 7 | 37 | 2 | 31 | 49 | 17 |
| **Polo 2020** | PDA | NR | NR |  | NR | NR | NR | NR | NR | NR | NR | NR |
| **Prichard 2013** | PDA | 58 | 61 |  | NR | NR | NR | NR | NR | NR | NR | NR |
| **Prieto-Velasco 2015** | PDA | 59.9* |  | 66.5, 55.2–76.5 * | NR | NR | NR | NR | NR | NR | NR | NR |
| **Prieto-Velasco 2015** | Control | 59.9* |  | 66.5, 55.2–76.5 * | NR | NR | NR | NR | NR | NR | NR | NR |
| **Vandemheen 2009** | PDA | 64 | 30.1, 9 |  | NR | NR | NR | NR | 4 | 44 | 51 | NR |
| **Vandemheen 2009** | Control | 46 | 30.7, 9 |  | NR | NR | NR | NR | 4 | 41 | 56 | NR |
| **Volk 2014** | PDA | 55 |  | 56, 20-67 | 4 | 7 | 2 | 87 | NR | NR | NR | NR |
| **Waterman 2018** | PDA | 56 | 55.8, 11.2 |  | 71 | 0 | 0 | 29 | 20 | 46 | 34 | NR |
| **Waterman 2018** | Control | 59 | 51.1, 13 |  | 82 | 0 | 0 | 18 | 22 | 33 | 45 | NR |
| **Waterman 2019** | PDA | 52 |  | 54,10 | 72 | 0 | 0 | 28 | NR | 48^+^ | 52 | NR |
| **Waterman 2019** | PDA (educator guided) | 49 |  | 54,12 | 70 | 0 | 0 | 30 | NR | 57^+^ | 43 | NR |
| **Waterman 2019** | Control | 52 |  | 53,10 | 71 | 0 | 0 | 29 | NR | 54^+^ | 46 | NR |
| **Waterman 2020** | PDA | 58.7 | 52.7, 13.0 |  | 27 | 38 | 2 | 34 | 2 | 31 | 56 | 12 |
| **Waterman 2020** | Control | 62.5 | 53.2, 13.3 |  | 23 | 40 | 1 | 36 | 3 | 33 | 52 | 12 |
| **Mean**  **Totals** |  | 57.7 | 53.0 |  | 52.5 | 8.6 | 7.1 | 34.4 | 7.4 | 48.6 | 41.8 | 13.7 |

*Table S6: Table detailing demographics of participants in the studies. NR Not Reported, PDA Patient Decision Aid, SD Standard Deviation, IQR Interquartile range. * these results were reported for the entire study, not broken down into PDA and control, the overall cohort demographics have been reported here.* ^+^ Notates when less than 8^th^ grade was not reported so this incorporates those educated to high school or less.

| Study Identifier | How PDA can be accessed if available | How was it delivered | Format of PDA | Where in the pathway of care |
| --- | --- | --- | --- | --- |
| Axelrod 2017 | https://www.mytransplantcoach.org/#/ | Patient directed | Online website, phone application | Prior to initial transplant assessment |
| Boulware 2018 | NA * | Patient directed | DVD, handbook. | Once patients had started dialysis. Some were already waitlisted for kidney transplant |
| Dubin 2019 | Behind paywall, https://www.crickethealth.com/kidney-care-providers/ | Patient directed | Online website | Between eGFR 10-30 ml/min/1.73m2 i.e pre-dialysis with Chronic kidney disease. |
| Gordon 2017 | https://informme.cbits.northwestern.edu/system/ | Patient directed | iPad application and online | After routine transplant education and clinic visit. Either for transplant evaluation or re-evaluation |
| Kayler 2020 | https://youtu.be/SOAQ8uWtrws https://www.youtube.com/watch?v=nOd3IakH_xA&feature=youtu.be | Patient directed | Online video | At time of transplant evaluation |
| Mucsi 2018 | https://etontario.org/ not accessible to public. * | By research assistants and dialysis nurses, as well as by patients alone. | Videos, handouts | Patients on maintenance dialysis and their potential living donors |
| Patzer 2018 | [https://ichoosekidney.emory.edu/, or via phone application in certain countries](https://ichoosekidney.emory.edu/,%20or%20via%20phone%20application%20in%20certain%20countries) | By physician during consultation | Online website, phone application | At evaluation for transplantation |
| Polo 2020 | NA | By physician during consultation | Online website | Patients with advanced cystic fibrosis |
| Prichard 2013 | www.optiongrid.org/resources/chronickidneydisease_evidence.pdf | By CKD Nurse educators during in home education | Paper | When the patient had an eGFR < 20 ml/min/1.73m2but had not yet been educated |
| Prieto-Velasco 2015 | NA | By nephrologist and an education-skilled nurse, potentially with other allied health staff | Electronic software, paper, DVD | Prior to commencing dialysis |
| Vandemheen 2009 | http://decisionaid.ohri.ca/decaids.html. | Patient directed | Online, paper | Before referral to lung transplant after transplant education |
| Volk 2014 | NA | Patient directed | Online | Already been reviewed and educated about risk of liver transplantation |
| Waterman 2018 | NA | Transplant educators, supported by videos and brochures | Video, printed information. | For all eligible dialysis patients, some were already active on the transplant list |
| Waterman 2019 | NA | Patient directed | DVD, paper, text messages | After starting dialysis |
| Waterman 2020 | NA | Tailored electronic report, handbook, DVD | Tailored electronic report, handbook, DVD | When presenting for transplant evaluation. |

*Table S7: table describing format and administration of PDA. * authors provided reviewers’ access. NA not available. DVD Digital video disc. eGFR estimate glomerular filtration rate*

| **Study Identifier** | **Name** | **Decision** | **Description of health problem*** | **Explicit description of the decision*** | **Values clarification *** | **Options, benefits, harms, consequences*** | **Guidance in communication** | **Guidance in deliberation** | **Intervention numerical probabilities** | **Personal stories** | **Reading level or other strategies to help understanding** | **Tailoring of information or probabilities** |
| --- | --- | --- | --- | --- | --- | --- | --- | --- | --- | --- | --- | --- |
| **Axelrod 2017** | My Transplant Coach | Kidney Transplant vs Dialysis | Y | Y | Y | Y | Y | Y | Y | Y | Y | Y |
| **Boulware 2018** | PREPARED | Kidney Transplant vs Dialysis | Y | Y | Y | Y | Y | Y | Y | Y | Y | N |
| **Dubin 2019** | Modality Decision Program | Kidney Transplant vs Dialysis | Y | Y | Y | Y | Y | NR | NR | Y | Y | N |
| **Gordon 2017** | Inform Me | Kidney Increased Viral Risk Donor | Y | Y | Y | Y | N | N | Y | Y | Y | N |
| **Kayler 2020** | Simplify KDPI | Different KDPI kidney | Y | Y | Y | Y | N | N | Y | N | Y | N |
| **Kayler 2020** | IRD-1-2-3 | Kidney from Increased Viral Risk Donor | Y | Y | Y | Y | N | N | Y | N | Y | N |
| **Mucsi 2018** | Explore Transplant Ontario | Kidney Transplant vs Dialysis | Y | Y | Y | Y | N | Y | Y | Y | NR | N |
| **Patzer 2018** | iChoose Kidney | Kidney Transplant vs Dialysis | Y | Y | Y | Y | N | N | Y | N | N | Y |
| **Polo 2020** | Informed Choices Cystic Fibrosis Decision Aid | Lung transplant | Y | Y | Y | Y | N | Y | Y | Y | Y | Y |
| **Prichard 2013** | Chronic Kidney Disease: Option Grid | Kidney Transplant vs Dialysis | Y | Y | Y | Y | N | N | N | N | N | N |
| **Prieto-Velasco 2015** | Education Process | Kidney Transplant vs Dialysis | Y | Y | Y | Y | Y | Y | NR | Y | NR | N |
| **Vandemheen 2009** | Lung Transplantation in Patients with Cystic Fibrosis | Lung transplant | Y | Y | Y | Y | Y | Y | Y | N | Y | N |
| **Volk 2014** | Liver Quality Decision Aid | Livers with different graft survival. | Y | Y | Y | Y | Y | NR | NR | NR | Y | Y |
| **Waterman 2018** | Explore Transplant | Kidney Transplant vs Dialysis | Y | Y | Y | Y | Y | Y | N | Y | Y | N |
| **Waterman 2019** | Explore Transplant at Home | Kidney Transplant vs Dialysis | Y | Y | Y | Y | NR | Y | NR | Y | Y | N |
| **Waterman 2020** | Your Path to Transplantation | Kidney Transplant vs Dialysis | Y | Y | Y | Y | NR | Y | NR | Y | NR | Y |

Table S8: SUNDAE checklist table. Y Yes, N No, NR Not available. * required to be considered a decision aid

| **Proportion of patients who felt PDA was accepable** | | | |
| --- | --- | --- | --- |
| **Study Identifier** | **PDA % (n)** | **Control** | **Note** |
| Axelrod et al. | 86% (70) |  |  |
| Bouleware et al. | 73% (16) |  |  |
| Dubin et al. | 100% (25) |  |  |
| Kayler et al. | 97% (41) |  |  |
| Vandeheem et al. | 47% (33) | 35% | p=0.01 |
| Waterman et al 2020. | 95% (387) |  |  |

*Table S9: Proportions of patients who felt PDA was acceptable*

| **Proportion who felt informed** | | | | | |
| --- | --- | --- | --- | --- | --- |
| **Study Identifier** | **Kidney Transplant Type** | **Control after %** | **PDA before %** | **PDA after %** | **Notes** |
| [Axelrod 2017](applewebdata://A3936DE9-066C-42BC-B00B-AFADF8161AF7/#_ENREF_20) | LD |  | 27 | 52 | p <0.001 |
|  | DD |  | 40 | 72 | p <0.001 |
| [Waterman 2018](applewebdata://A3936DE9-066C-42BC-B00B-AFADF8161AF7/#_ENREF_32) | LD | 51 |  | 84 | p <0.001, OR 5.67 (3.2-10.1) |
|  | DD | 51 |  | 80 | p <0.001, OR 4.09 (2.68-6.21) |
| [Waterman 2018](applewebdata://A3936DE9-066C-42BC-B00B-AFADF8161AF7/#_ENREF_32) | LD | 73 |  | 92 | p<0.001 |
|  | DD | 70 |  | 84 | p=0.0003 |

*Table S10: Proportion of patients who reported feeling informed to make a decision about LD or DD kidney transplantation. LD Living Donor. DD deceased donor*

*
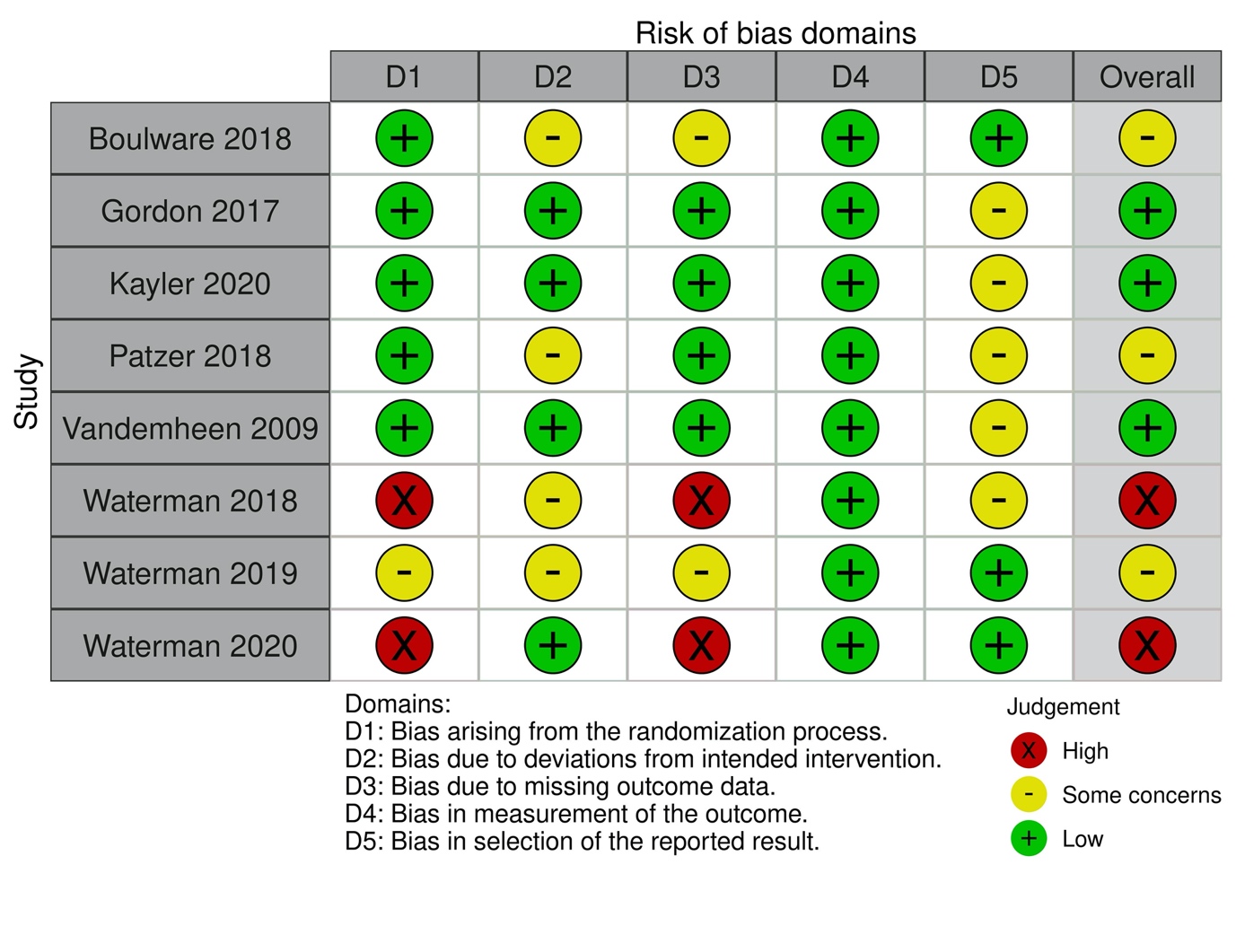
*

*Figure S1: Traffic light plot for Risk of Bias 2^62^ domains for all RCT studies included in the review for the outcome of knowledge (study by study). Boulware et. al did not assess knowledge, and therefore risk of bias was based on the primary outcome which was actions to pursue transplant. ^67^*

*
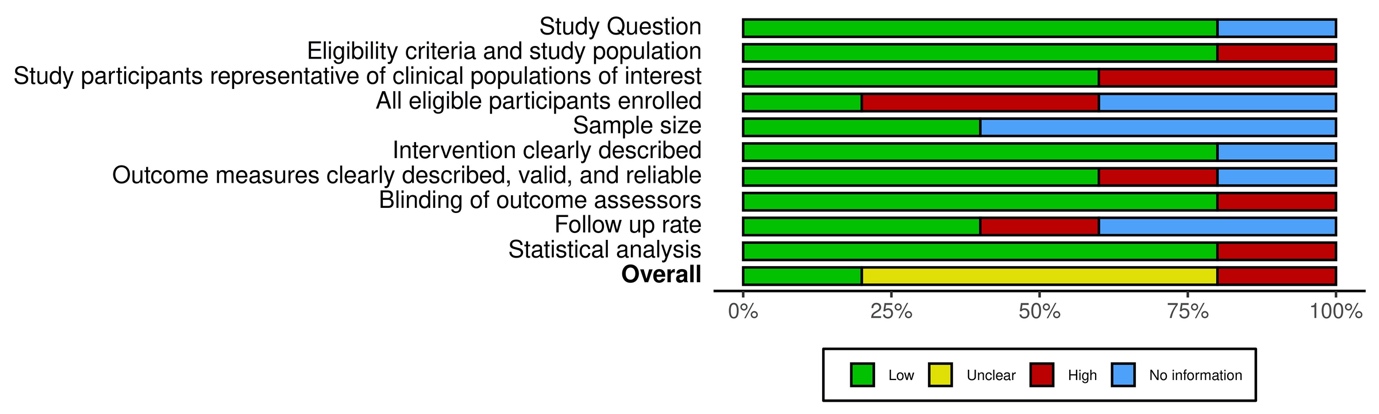
*

*Figure S2: Summary plot of National Institutes of Health risk of bias domains for pre-test post-test studies (by domains).*


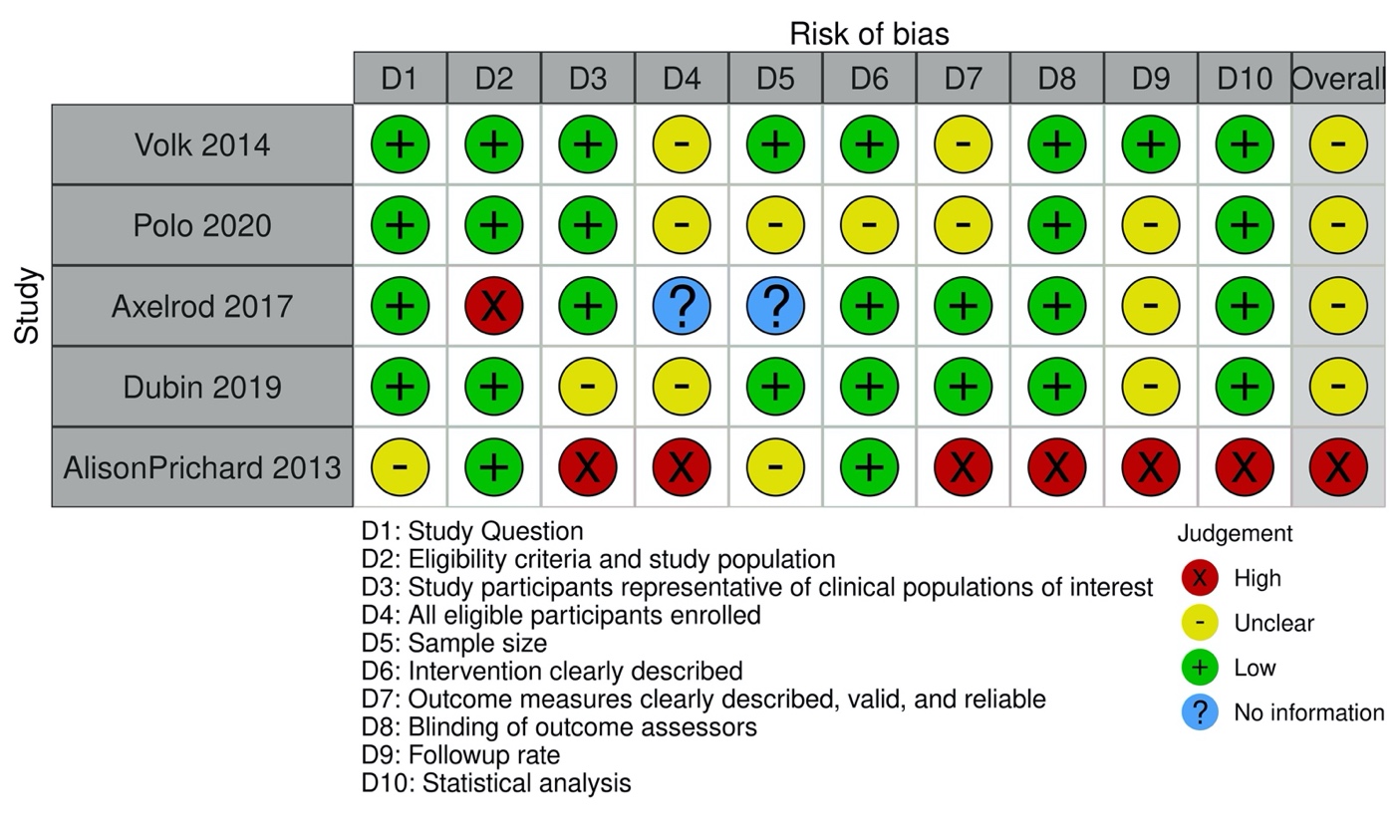


*Figure S3: Traffic light plot National Institutes of Health risk of bias domains for all pre-test post-test studies (study by study).*

*
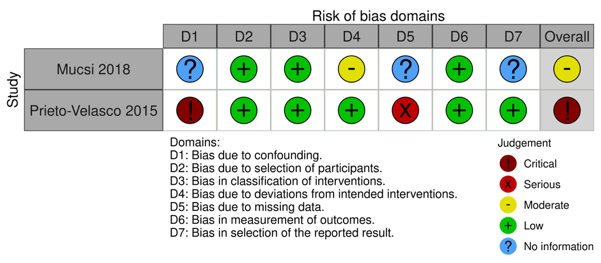
*

*Figure S4: Traffic light plot for the outcome of knowledge (study by study) for non-randomised studies using ROBINS I^63^ risk of bias tool*


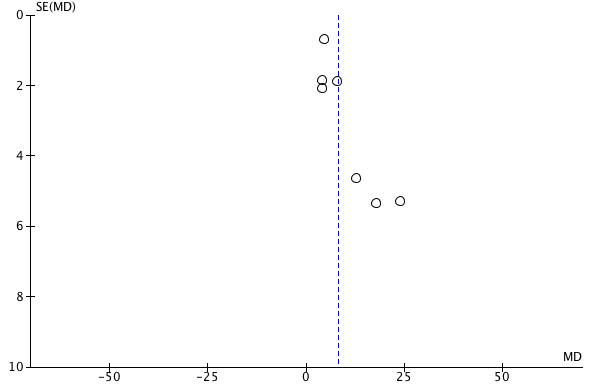


*Figure S5: Forest plot assessing for reporting bias for the outcome of knowledge for all RCT included in the meta-analysis.*
